# Protective Effect of Quercetin on Posttraumatic Cardiac Injury

**DOI:** 10.1038/srep30812

**Published:** 2016-07-29

**Authors:** Zehao Jing, Zhuorun Wang, Xiujie Li, Xintao Li, Tingting Cao, Yue Bi, Jicheng Zhou, Xu Chen, Deqin Yu, Liang Zhu, Shuzhuang Li

**Affiliations:** 1 Dalian Medical University, Department of Physiology, Dalian, 116044, China

## Abstract

Quercetin is an important dietary flavonoid present in fruits and vegetables and has attracted attention because of its anti-inflammatory and anti-oxidative properties. Inflammation and oxidative stress play important roles in posttraumatic cardiomyocyte apoptosis, which contributes to secondary cardiac dysfunction. This study investigates the protective effect of quercetin on trauma-induced secondary cardiac injury and the mechanisms involved. Widely accepted nonlethal mechanical trauma models were established. *In vivo*, cardiomyocyte apoptosis and cardiac dysfunction in rats were assessed using TUNEL staining and a biological mechanic experiment system. *In vitro*, cell viability, tumour necrosis factor-α (TNF-α), reactive oxygen species (ROS) and [Ca^2+^]_i_ of H9c2 cells were detected using an MTT assay, ELISA, and 2′,7′-dichlorofluorescin diacetate and fluo-4 acetoxymethyl ester assays respectively. Quercetin pretreatment (20 mg/kg i.p.; 0.5 h before trauma) significantly improved posttraumatic cardiomyocyte apoptosis and cardiac dysfunction. Pretreatment with quercetin (20 μM; 24 h before trauma plasma addition) significantly attenuated trauma-induced viability decreases, TNF-α increases, ROS overproduction and [Ca^2+^]_i_ overload in H9c2 cells. In conclusion, quercetin may reverse posttraumatic cardiac dysfunction by reducing cardiomyocyte apoptosis through the suppression of TNF-α increases, ROS overproduction and Ca^2+^ overload in cardiomyocytes, representing a potential preventive approach for the treatment of secondary cardiac injury after mechanical trauma.

Mechanical trauma (MT), such as that caused during motor vehicle crashes, athletic competition, and war, currently represents a primary economic and medical burden. Trauma has become the most common cause of death in people under the age of 45 years^1^,^2^. Several studies have indicated that MT can cause direct heart damage, such as coronary artery dissection^3^,^4^. As a result of advanced prehospital care and regional trauma system development, fewer seriously injured patients are dying at the scene of an accident. However, several recently published clinical reports have suggested that nonfatal MT can induce secondary cardiac dysfunction even in the absence of direct cardiomyocyte injury during the first 24 h^5^^,^^6^^,^^7^^,^^8^.

Secondary cardiac dysfunction can present as many severe cardiac diseases, such as myocardial infarction^9^, severe congestive heart failure^8^, traumatic ventricular septal defect (VSD)^10^, and even multiple organ dysfunction syndrome (MODS), which has detrimental effects on human health and living standards^11^. Secondary cardiac dysfunction is difficult to diagnose because the clinical presentation, course and severity are variable^12^. In addition, the primary traumatic injury may obscure the typical chest pain associated with cardiac ischaemia^13^ and the mechanism has never been elucidated. Thus, the mechanism of secondary cardiac dysfunction needs to be investigated.

Our previous study^14^ demonstrated that trauma-induced overproduction of TNF-α is the main reason for cardiomyocyte apoptosis, which contributes to posttraumatic cardiac dysfunction. Moreover, we have provided clear evidence that TNF-α initiates cardiomyocyte apoptosis by the overproduction of cytotoxic reactive oxygen/nitrogen species in cardiomyocytes. These results support the hypothesis that the overproduction of TNF-α and ROS may be the primary cause of secondary cardiomyocyte apoptosis, which eventually leads to secondary cardiac dysfunction after MT. Therefore, developing drugs with anti-inflammatory and anti-oxidative properties may have profound significance for patients who have experienced MT.

Quercetin is a natural flavonoid compound that is widely distributed in fruits and vegetables. It exerts numerous beneficial effects on neuroprotective, anti-inflammatory, anti-ischaemic, antimutagenic, antiviral and cardiovascular protection processes^15^,^16^,^17^,^18^,^19^,^20^. Many studies have shown that quercetin acts as a novel protectant by mitigating the increased levels of TNF-α and ROS^21^,^22^,^23^,^24^. Given its protective properties and therapeutic potential, we speculated that quercetin may have a protective effect against MT-induced cardiomyocyte apoptosis. To test our hypothesis and explore its mechanism, *in vivo* and *in vitro* experiments were undertaken. Changes in cardiac function, cell viability, and TNF-α, ROS and [Ca^2+^]_i_ levels with or without quercetin treatment were observed and analysed.

## Results

### MT induced cardiomyocyte apoptosis

**Figure 1:**
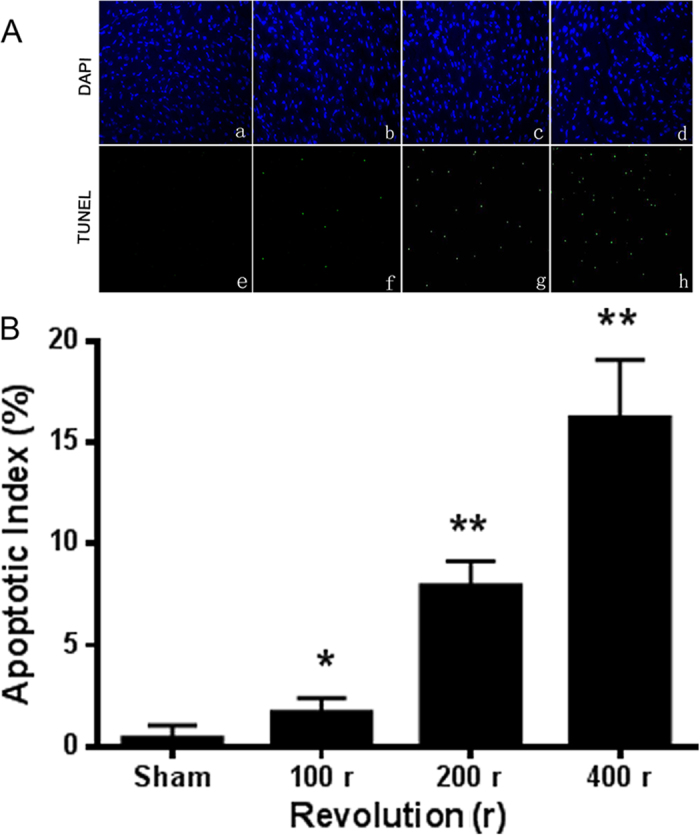
MT mediated cardiomyocyte apoptosis. (**A**) Traumatic injury caused cardiomyocyte apoptosis for 0 r (a,e), 100 r (b,f), 200 r (c,g) and 400 r (d,h) (n = 5). Total nuclei (a–d) were determined using DAPI staining (blue), and apoptotic nuclei (e–h) were identified using positive TUNEL staining (green). (**B**) The apoptotic index was calculated by counting TUNEL signals in 5 randomly selected crypts after TUNEL staining, as in A. Statistical comparisons were performed using one-way analysis of variance. *P < 0.05, **P < 0.01 vs. sham.

To determine the most appropriate trauma intensity in the nonlethal MT model, the cardiomyocyte apoptosis states of rats subjected to 0 r, 100 r, 200 r, and 400 r were assessed using TUNEL and DAPI staining 12 h after trauma. Figure 1A shows the results of DAPI (a, b, c, d) and TUNEL (e, f, g, h) staining, which indicated the density of cardiomyocytes (a, b, c, d are of the same intensity) and the degree of apoptosis. There were nearly no TUNEL-positive nuclei detected in the samples isolated from rats subjected to sham trauma. However, the cardiomyocytes of rats subjected to 100 r, 200 r and 400 r showed significant apoptosis (Fig. 1B). As the mortality rate of the rats in the 400 r group (30%) was significantly higher than that of the rats in the 200 r (0%) and 100 r (0%) groups, we chose 200 r as the standard number of revolutions in the nonlethal MT model to reduce mortality while obtaining the greatest apoptosis effect after trauma.

### Quercetin decreased MT-induced cardiomyocyte apoptosis

**Figure 2:**
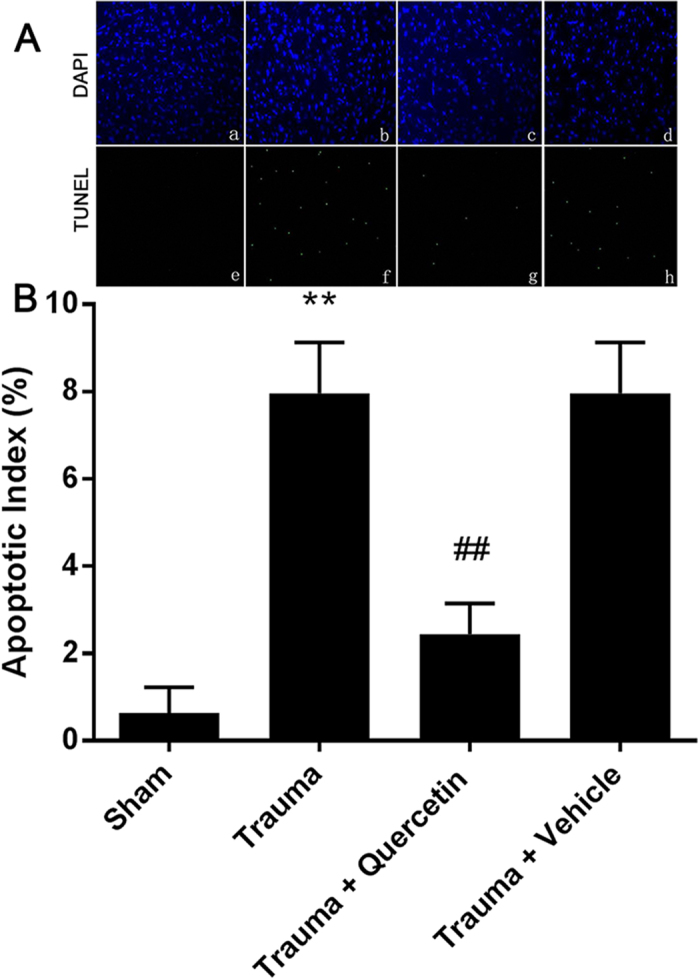
Quercetin attenuated cardiomyocyte apoptosis. (**A**) Representative images of the TUNEL assay (n = 5). Significant differences were observed between trauma vs. sham and trauma vs. trauma + quercetin. Quercetin was used at 20 mg/kg i.p. 0.5 h before trauma. (**B**) The apoptotic index was calculated by counting TUNEL signals in 5 randomly selected crypts after TUNEL staining, as in A. Statistical comparisons were performed using one-way analysis of variance. **P < 0.01 vs. sham; ^##^P < 0.01 vs. trauma.

To test the protective effect of quercetin on MT-induced cell apoptosis *in vivo*, rats with MT (200 r) were pretreated with quercetin or vehicle (20 mg/kg i.p.; 0.5 h before trauma). The dose for quercetin (20 mg/kg) was based on the dose used in previously published articles^25^,^26^. TUNEL and DAPI staining were performed as above (Fig. 2A). The apoptotic cardiomyocytes of posttraumatic rats increased significantly, whereas quercetin significantly reduced the number of apoptotic cells. However, the changes in the degree of apoptosis caused by pretreatment with vehicle were not statistically significant (Fig. 2B). Taken together, these data indicated that quercetin attenuated MT-induced cardiomyocyte apoptosis.

### Quercetin improved cardiac dysfunction

**Figure 3:**
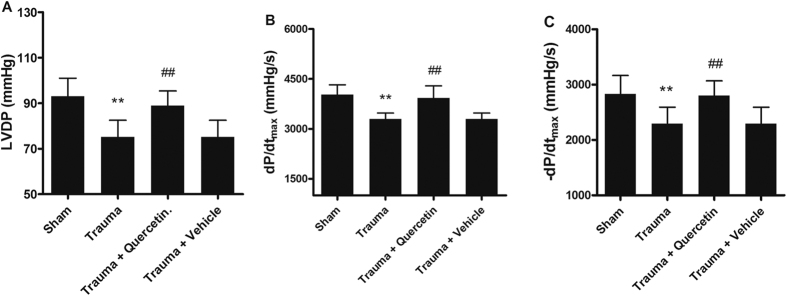
Nonlethal traumatic injury caused cardiac dysfunction, and quercetin alleviated trauma-induced cardiac dysfunction. The cardiac haemodynamic parameters of the control, trauma, trauma + quercetin and trauma + vehicle group for 200 r were recorded 12 h after trauma. Mechanical trauma resulted in a significant decrease in LVDP (**A**), +*dp*/*dtmax* (**B**) and −*dp*/*dtmax* (**C**), which was effectively prevented by pre-treatment with quercetin. Statistical comparisons were performed using one-way analysis of variance. n = 5 rats/group, **P < 0.01 vs. sham; ^##^P < 0.01 vs. trauma.

Evaluation of myocardial function is the basis for managing heart disease. Left ventricular developed pressure (LVDP, Fig. 3A), peak rates of rise in the first derivative of the left ventricular pressure (+*dp*/*dtmax*, Fig. 3B), and peak rates of fall in the first derivative of the left ventricular pressure (−*dp*/*dtmax*, Fig. 3C) were continuously monitored on a recorder to evaluate cardiac function. Figure 3A–C showed cardiac dysfunction with a lower LVDP, reduced +*dp*/*dtmax* and lessened −*dp*/*dtmax* compared with each control group, respectively. While quercetin attenuated this downtrend significantly, vehicle treatment did not. These results demonstrated that quercetin effectively improved MT-induced cardiac dysfunction.

### Appropriate concentration of quercetin for H9c2 cells

**Figure 4:**
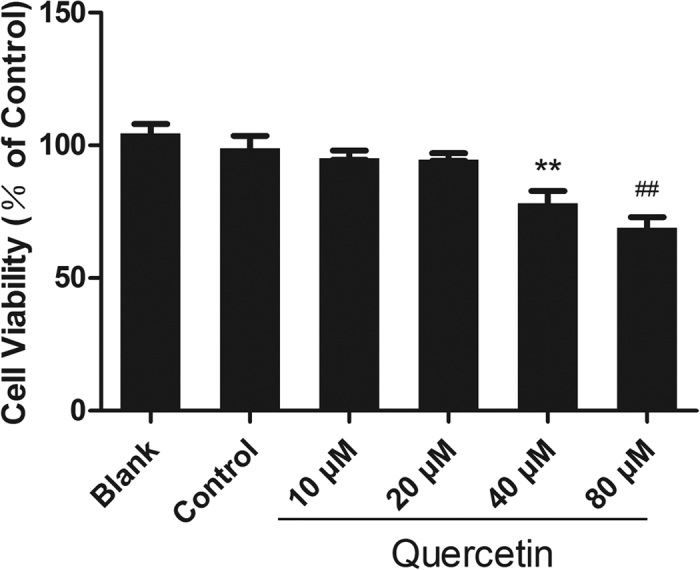
The viability of H9c2 cells was influenced by the quercetin concentration. The viability of H9c2 cells induced by quercetin at various concentrations was detected using an MTT assay. Quercetin showed no cytotoxicity at low concentrations (10 μM, 20 μM), but it decreased cell viability at 40 μM and 80 μM. Statistical comparisons were performed using one-way analysis of variance. n = 3 per group, **P < 0.01, ^##^P < 0.01 vs. control.

To determine the most appropriate quercetin concentration, 10 μM, 20 μM, 40 μM, or 80 μM quercetin was added to H9c2 cells, and the cells were analysed using the MTT assay. As shown in Fig. 4, there was no statistically significant difference in cell viability when the concentrations of quercetin were 10 μM or 20 μM compared with the control group (with vehicle) and blank group (without vehicle), indicating that quercetin had no toxicity at low concentrations. Cell viability decreased when the concentration of quercetin was 40 μM and significantly decreased at 80 μM compared with the control group, indicating that quercetin had toxicity at these two concentrations. Thus, concentrations under 20 μM may be safe for H9c2 cells.

### Quercetin protected TP-induced H9c2 cells

**Figure 5:**
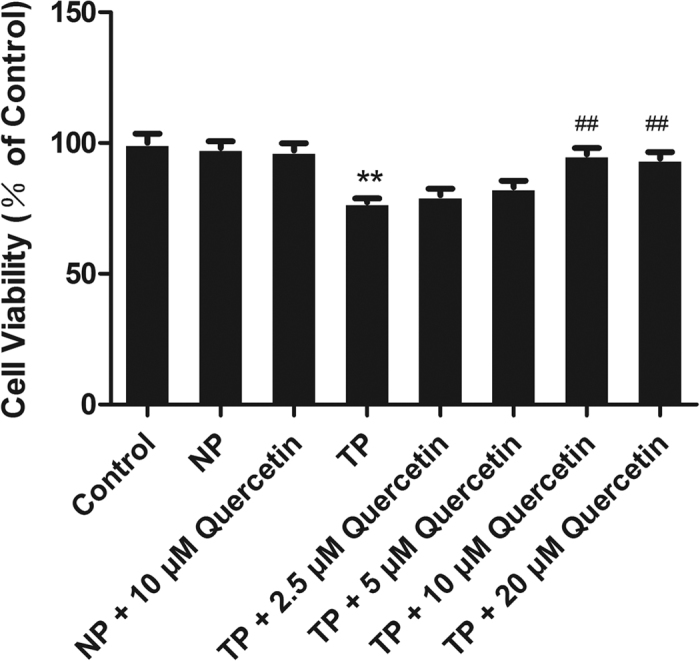
Quercetin reversed TP-induced cell viability reductions in H9c2 cells. The viability of H9c2 cells was detected using an MTT assay and was significantly reduced following TP treatment; the decreased viability was attenuated by pre-treatment with quercetin. The effect of quercetin at 2.5, 5, 10, or 20 μM was detected using an MTT assay, which proved that 10 μM was the best concentration to increase cell viability after TP treatment. Statistical comparisons were performed using one-way analysis of variance. n = 3 per group, **P < 0.01 vs. control; ^##^P < 0.01 vs. TP.

TP (50% V/V)-induced cells *in vitro* is a simple model of organ MT, at least partly reflecting the pathophysiology *in vivo*. To simulate *in vivo* nonlethal mechanical trauma, H9c2 cells were exposed to TP. To determine whether quercetin has a protective effect on TP-induced H9c2 cells, quercetin (2.5 μM, 5 μM, 10 μM, or 20 μM) was added 24 h in advance. Cell viability was detected using an MTT assay. The cell viability of H9c2 cells induced by TP notably decreased compared with that induced by normal plasma (NP), while previous treatment with quercetin significantly inhibited the decrease. As Fig. 5 shows, 10 μM quercetin was the most effective at inhibiting TP-induced viability decreases. Accordingly, the NP + 10 μM group was added to the study to rule out the toxicity of quercetin at the concentration of 10 μM in NP.

### Quercetin reduced circulating TNF-α level

In our previous study^27^, cardiomyocytes were cultivated with cytomix (a mixture of IFN-γ, IL-1β, and TNF-α) and with IFN-γ, IL-1β, or TNF-α alone. The caspase-3 activation of cardiomyocytes incubated with cytomix and TNF-α was much higher than that with normal plasma. In addition, the exposure of normal cardiomyocytes to TP revealed apparent cell apoptosis; this phenomenon was virtually abolished through the incubation of TP with anti-TNF-α or TP isolated from TNF-α−/− mice, indicating that TNF-α plays a critical role in cardiac injury after MT.

**Figure 6:**
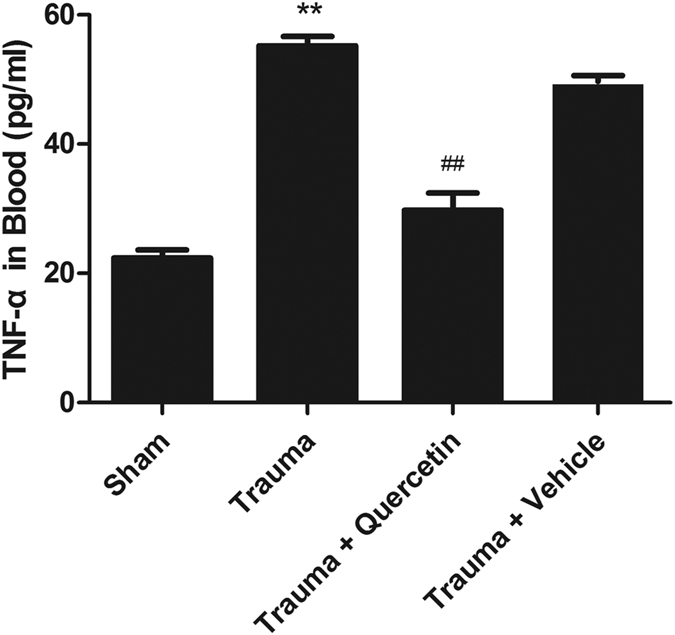
Quercetin decreased the TNF-α concentration in the circulation. TNF-α in the peripheral circulation system was apparently increased 1.5 h after trauma, which was significantly attenuated by pretreatment with quercetin (20 mg/kg i.p. 0.5 h before trauma). Statistical comparisons were performed using one-way analysis of variance. n = 5 rats/group, **P < 0.01 vs. sham; ^##^P < 0.01 vs. trauma.

As the amount of TNF-α in the circulation system of posttraumatic rats peaked at 1.5 h after trauma (2 h ahead of significant cardiomyocyte apoptosis), ELISA kits were used to determine the amount of TNF-α in the circulatory system 1.5 h after MT in the four groups (sham group, trauma group, trauma + quercetin group and trauma + vehicle group). As shown in Fig. 6, with quercetin pretreatment, overproduction of TNF-α was significantly inhibited, whereas there was no apparent change in TNF-α in the trauma + vehicle group compared with the trauma group.

### Quercetin suppressed ROS overproduction in H9c2 cells

**Figure 7:**
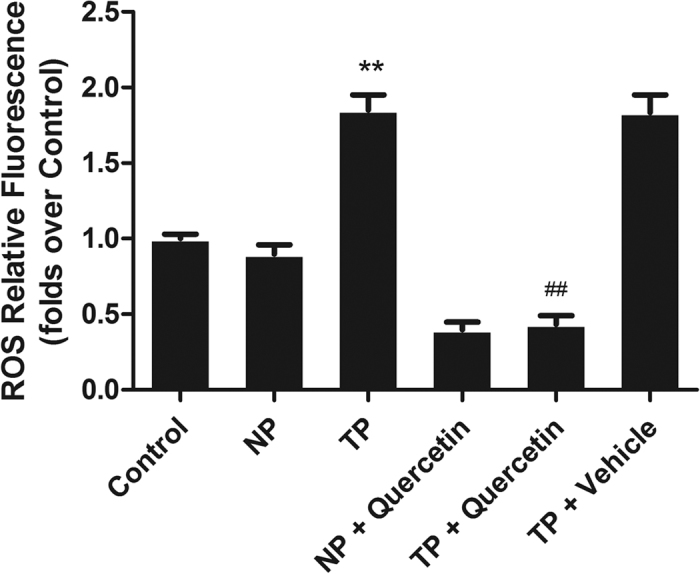
Quercetin ameliorated H9c2 cell oxidative stress. Intracellular ROS production was determined using DCFH-DA. H9c2 cell oxidative stress was initiated by TP and was significantly attenuated by pretreatment with quercetin (10 μM). Statistical comparisons were performed using one-way analysis of variance. n = 3 per group, **P < 0.01 vs. control; ^##^P < 0.01 vs. TP.

Oxidative stress was proven in our previous study to play an important role in MT-induced cardiomyocyte apoptosis. Therefore, we examined the protective effect of quercetin on TP-induced oxidative imbalance in H9c2 cells. As shown in Fig. 7, the intracellular ROS level of TP-treated cells showed a remarkable increase, while quercetin scavenged excess ROS to protect cells at the concentration of 10 μM in the presence of TP. Interestingly, pretreatment with quercetin in NP significantly decreased intracellular ROS in H9c2 cells, indicating that quercetin could eliminate ROS both in NP and TP.

### Quercetin inhibited Ca^2+^ in H9c2 cells

**Figure 8:**
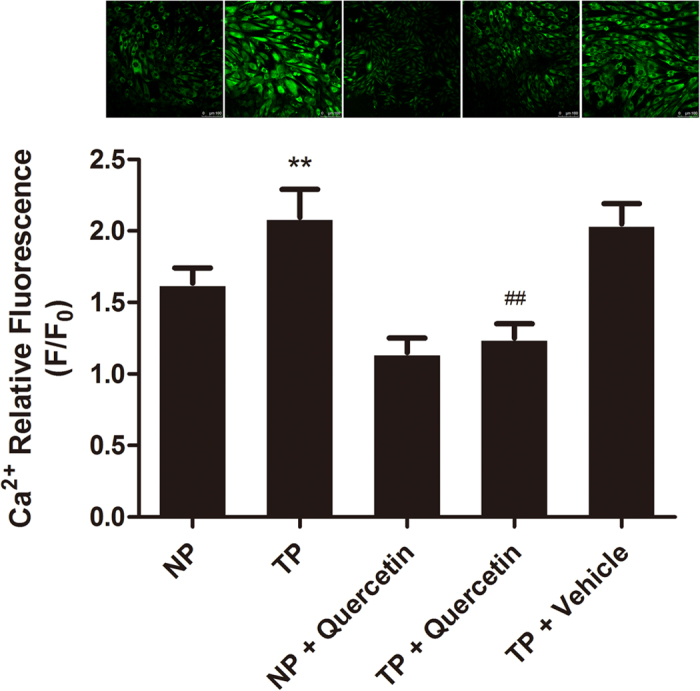
Quercetin reduced TP-initiated Ca^2+^ overload in H9c2 cells. TP initiated Ca^2+^ overload in H9c2 cells, and the incubation of H9c2 cells with quercetin (10 μM) facilitated an apparent decrease in intracellular Ca^2+^ release. Statistical comparisons were performed using one-way analysis of variance. n = 3 per group, **P < 0.01 vs. NP; ^##^P < 0.01 vs. TP.

Given the important role of cellular Ca^2+^ in apoptosis and necrosis^28^, MT-initiated Ca^2+^ overload in H9c2 cells was measured. As shown in Fig. 8, TP significantly enhanced the level of [Ca^2+^]_i_, and quercetin pretreatment for 2 min suppressed this change, providing evidence that MT-initiated [Ca^2+^]_i_ overload may contribute to posttraumatic cell apoptosis that can be relieved by quercetin.

## Discussion

In the present study, we have demonstrated the protective effects of quercetin on posttraumatic cardiac injury, the mechanism of which is mediated by reducing cell apoptosis through the inhibition of inflammation and oxidative stress and attenuating intracellular Ca^2+^ overproduction in cardiomyocytes.

Many previous studies have investigated the effect of flavonoids in other post-traumatic injury models^25^,^26^. Xu *et al*. found that luteolin protected the mouse brain from traumatic brain injury by inhibiting the inflammatory response and that luteolin-induced autophagy may play a pivotal role in neuroprotection. Itoh *et al*. demonstrated the neuroprotective effect of (−)-epigallocatechin-3-gallate in rats when administered pre- or post-traumatic brain injury. However, the post-traumatic injury models used in those studies were different from those used in our study in terms of the regions involved and the methods used to produce trauma. To the best of our knowledge, this study is the first to examine the protective effect of quercetin on MT-induced cardiac injury.

Mechanical trauma, even nonlethal mechanical trauma not causing death at the moment of an accident, can lead to secondary organ dysfunction, which is characterized by a lack of circulatory shock and no direct cardiac injury^11^. To detect the protective effect of quercetin on secondary cardiac dysfunction, a well-accepted nonlethal traumatic model was created *in vivo*. As shown in Figs 1, 2, 3, rats subjected to trauma (200 r) exhibited significant cardiomyocyte apoptosis and decreased cardiac function; these trends were reversed by pretreatment with quercetin (20 mg/kg i.p.; 0.5 h before trauma), strongly supporting the hypothesis that quercetin can effectively protect against MT-induced cardiomyocyte apoptosis and alleviate secondary cardiac dysfunction.

The dose for quercetin (20 mg/kg) was established based on available articles^25^,^26^. The levels of quercetin in plasma following intraperitoneal administration (50 mg/kg) in Sprague–Dawley rats were detected by Mauro Piantelli *et al*.^29^ in a published article. As described in their study, the highest plasma concentrations were found at the earliest sampling time, approximately 15 min after dosing. The maximal concentration is (0.081 + 0.34) μg/ml, which is much less than the minimum toxic dose detected in our study (40 μM/L approximately equalling 12 μg/ml). Thus, it is safe to use quercetin at the concentration of 20 mg/kg (<50 mg/kg).

As shown in Fig. 4, to determine the most appropriate concentration of quercetin *in vitro*, 10 μM, 20 μM, 40 μM, or 80 μM quercetin was added to H9c2 cells; the cells were analysed using an MTT assay, which proved that quercetin decreased cell viability at the concentrations of 40 μM and 80 μM but not at 10 μM and 20 μM. The reason for this cytotoxic effect has never been elucidated. It has been proven that, owing to its specific planar chemical structure, quercetin readily forms chelates with metal ions and that these complexes of bioactive compounds and metal ions, such as lanthanum, often show cytotoxic properties in human cervical carcinoma cells and induce dose-dependent pro-oxidative effects^30^. Another study demonstrated that quercetin may act as a cytotoxic pro-oxidant after its metabolic activation to semiquinone and quinoidal products^31^. Our previous study showed that some antioxidants, such as procyanidin and curcumin, exhibit toxicity when cultured with H9c2 cells individually, resulting in excessive clearance of ROS in H9c2 cells. These results are in accordance with the idea that ROS are useful for the existence and proliferation of cells^32^.

*In vitro*, H9c2 cells were cultivated with TP to reflect the pathophysiology of trauma *in vivo*. It is shown in Figs 4 and 5 that 10 μM quercetin had no cytotoxicity with or without NP and could reverse TP-induced cell viability decreases to the maximum extent compared with 2.5, 5, or 20 μM, indicating that 10 μM quercetin can be used as a protective dose.

We have previously proven that TNF-α produced by injured peripheral tissues is the main reason for the overproduction of the intracellular ROS that initiate cardiomyocyte apoptosis following non-lethal mechanical trauma^14^. Given our previous study and the anti-inflammatory and anti-oxidative properties of quercetin, TNF-α in blood and ROS in H9c2 cells were detected with or without quercetin. As shown in Figs 6 and 7, TNF-α in blood and ROS in H9c2 cells were enhanced by TP and were decreased by pretreatment with quercetin. Several studies have demonstrated that quercetin inhibits lipid peroxidation effectively by scavenging free radicals and/or chelating transition metal ions^33^, results similar to those of our study, indicating that the anti-oxidative effect of quercetin may contribute to the protective effect on MT-induced cardiomyocyte apoptosis.

It has been reported that intracellular free Ca^2+^ is essentially involved in the mechanism of apoptosis^28^ and that Ca^2+^ and ROS release are two cross-talk events that are important in TNF-α-mediated apoptosis^34^. As shown in Fig. 8, [Ca^2+^]_i_ increased after TP treatment and decreased after pretreatment with quercetin, indicating that quercetin may attenuate the overproduction of [Ca^2+^]_i_ to inhibit cardiomyocyte apoptosis.

In the experiments with calcium overload in H9c2 cells, quercetin was added 2 min prior to their exposition to TP. In our previous study, quercetin added 30 min, 15 min, 5 min, and 2 min in advance was tested and showed a similar effect on calcium overload. Thus, we chose the shortest time as the standard.

In conclusion, our study demonstrated for the first time that quercetin can attenuate cardiomyocyte apoptosis and improve cardiac dysfunction induced by MT. Its anti-inflammatory, anti-oxidative and Ca^2+^ scavenging properties may contribute to this protective effect. Given its extensive distribution and the high incidence of nonlethal mechanical trauma, quercetin may become a promising agent for preventing posttraumatic cardiac injury in the future.

## Methods

### Materials

Quercetin (purity >98%) was purchased from Melone Pharmaceutical Co., Ltd. (Dalian, China). Dulbecco’s modified Eagle medium (DMEM) was purchased from Gibco BRL Co., Ltd. (Grand Island, NY, USA). H9c2 cells were obtained from American Type Culture Collection (ATCC,Manassas, VA, USA; CRL-1446). The DCFH-DA ROS Detection Kit was purchased from Beyotime Institute of Biotechnology (Nanjing, China). The TUNEL (terminal deoxynucleotidyl transferase-mediated dUTP nick-end labelling) apoptosis detection kit was purchased from Roche (Shanghai, China). 3-(4,5-Dimethyl-2-thiazolyl)-2,5-diphenyl-2-H-tetrazolium bromide (MTT), RPMI-1640 media, 10% heat-inactivated foetal bovine serum (FBS), Fluo-4/Am and all other chemicals were purchased from Sigma (USA). The TNF-α assay kit was purchased from Sangon (Shanghai, China). The modified Noble-Collip drum was obtained from the Department of Physiology, Dalian Medical University. The BL-420 biological and functional experimental system and pressure sensors were purchased from Taimeng Sciences and Technology Limited (Chengdu, China). The BX51 fluorescence microscope was purchased from Olympus Co. (Japan). The microplate reader was purchased from BioTek (VT, USA). The SP8 laser confocal microscope was purchased from Leica Co. (USA).

### Ethics statement

This study conformed to the Guide for the Care and Use of Laboratory Animals published by the US National Institutes of Health and the Guide for the Care and Use of Laboratory Animals’ protocol published by the Ministry of the People’s Republic of China (issued 3 June, 2004) and was approved by the Institutional Animal Care and Use Committee of Dalian Medical University. The methods were carried out in accordance with the approved guidelines.

### Animals and groups

A total of 120 healthy and clean adult male Sprague Dawley (SD) rats (body weight: 210 ± 20 g, from Dalian Medical University) were fed a standard laboratory diet and water ad lib. and were maintained at 22 °C under a constant 12-hour light-dark cycle. The animals were acclimated to their surroundings for 3 days before experimentation and were divided into four groups equally according to the random indicator method (30 in each): sham group, trauma group, trauma + quercetin group (quercetin and DMSO at the ratio of 10 mg to 1 ml, 20 mg/kg ip; 0.5 h before MT), and trauma + vehicle group (DMSO, the same volume as above ip; 0.5 h before MT).

### Nonlethal traumatic rat model

Noble-Collip drum exposure is a well-accepted traumatic model that results in whole-body nonpenetrative mechanical trauma. Briefly, after anesthetization with 10% chloral hydrate (300 mg/kg i.p.), the rats were placed in a Noble-Collip drum and were subjected to 5-min rotations (200 rotations at a rate of 40 r/min). The traumatic rats (for trauma, trauma + quercetin, trauma + vehicle groups) were injured when the wheel was rotated, while the sham trauma rats (for sham group) were taped on the inner wall of the drum to avoid traumatic injury. The nonlethal MT rat models used in this Noble-Collip drum experiment were characterized by the lack of circulatory shock, no direct cardiac injury, and a 100% 24 h survival rate.

### Determination of cardiomyocyte apoptosis by TUNEL staining

The hearts of traumatized rats were isolated 12 h after trauma for each group (5–7 in each). To determine cardiomyocyte apoptosis in a quantitative manner, the hearts were perfused first with 0.9% NaCl for 5 min and then with 4% paraformaldehyde in PBS (pH 7.4) for 20 min. Four longitudinal sections from the free wall of the left ventricle were cut and further fixed in 4% paraformaldehyde in PBS for 24 h at room temperature. Fixed tissues were embedded in a paraffin block, and two slides at 4- to 5-μm thickness were cut from each tissue block. Immunohistochemical procedures for detecting apoptotic cardiomyocytes were performed using an apoptosis detection kit according to the manufacturer’s instructions. After rinsing with PBS, the slides were coverslipped with mounting medium containing DAPI to permit total nuclei counting.

Using a ×20 objective, the tissue slide was digitally photographed using a QICAM-Fast Digital Camera mounted onto an Olympus BX51 fluorescence microscope. The total nuclei (blue) and TUNEL-positive nuclei (green) in each field were counted in five randomly chosen fields. The index of apoptosis (number of TUNEL-positive nuclei/total number of nuclei ×100) was calculated and exported to Microsoft Excel for further analysis.

### Assessments of cardiac function *in vivo*

At 12 h after trauma, a thin cannula was inserted into the left ventricle of the rats (anesthetized by pentobarbital sodium, 40 mg/kg) through the right common carotid and was then connected to a pressure transducer for the measurement of left ventricular pressure (LVP). With the pressure transducer connected to a biological mechanic experiment system (BL-420), left ventricular peak systolic pressure (LVSP), left ventricular end-diastolic pressure (LVEDP), left ventricular developed pressure (LVDP = LVSP – LVEDP), peak rates of rise in the first derivative of the left ventricular pressure (+*dp*/*dtmax*), and peak rates of fall in the first derivative of the left ventricular pressure (−*dp*/*dtmax*) were continuously monitored on a recorder.

### Preparation of plasma

Blood was harvested from rats 1.5 h after trauma. The rats were deeply anesthetized with chloral hydrate throughout the process. Cardiac puncture into the apex cordis was used to collect blood into Eppendorf tubes containing 1% heparin. The tubes were then centrifuged (3000 r, 20 min, 4 °C), and the supernatant (TP) was collected. NP was obtained from the rats without trauma in the same way as above. TP and NP were both stored at −80 °C.

### Cultivation of H9c2 cells

H9c2 cells were cultured in high-glucose DMEM supplemented with 10% foetal bovine serum, 4 mM L-glutamine, 100 units/ml penicillin and 0.1 mg/ml streptomycin at 37 °C in a humidified chamber of 95% air and 5% CO_2_ atmosphere. Cell culture media were changed every 2–3 days, and the cells were sub-cultured once they reached 70–80% confluence. Cells between passages 7 and 13 were used in the experiments.

### Most appropriate concentration of quercetin detected by MTT

A 100-μl H9c2 cell suspension was loaded into each well of a 96-well plate and was cultured in the logarithmic growth phase until the cell concentration reached 5 × 10^7^/L. The cells were then treated with quercetin at a series of diluted concentrations (80, 40, 20, or 10 μM, respectively), except the cells in the blank group (without vehicle) and control group (with vehicle) for 24 h. Before harvesting, the cells were incubated with 20 μl of MTT in medium for 4 h at 37 °C. The viable cells converted the MTT to a blue-purple colour after dissolving in 150 μl of dimethyl sulfoxide (DMSO). The absorbance at 570 nm was measured using a microplate reader. All experiments were carried out in triplicate. Cell viability (%) = [(absorbance of cells treated with quercetin)/absorbance of control cells] ×100%.

### Protective effect of quercetin on H9c2 cells as detected by MTT

H9c2 cells were prepared in the same way until the concentration reached 5 × 10^7^/L. The cells were divided into a control group, NP group (pretreatment with NP, 50% V/V), TP group (pretreatment with TP, 50% V/V), TP + 2.5 μM quercetin group, TP + 5 μM quercetin group, TP + 10 μM quercetin group and TP + 20 μM quercetin group (pretreatment with quercetin, 2.5/5/10/20 μM; TP added 24 h later). We added the NP + 10 μM quercetin group after 10 μM was proven to be the best concentration for protecting cells from TP-induced viability decrease. After processing as described, all of the cells were cultured for 3 h before the MTT assay was performed as above.

### Determination of circulating TNF-α level

To determine the circulating TNF-α level of the sham group, trauma group, trauma + quercetin group and trauma + vehicle group quantitatively, we used prepared animal serum and the TNF-α assay kit for rats. The blank, standard and specimen samples were set in triplicate. After adding the samples following the instruction manual, the plates were closed with the membrane and incubated for 60 min at 37 °C. Next, the plates were washed 5 times, the chromogenic agent was administered, and then the plates were incubated for another 5–15 min. Finally, after adding stop solution, we acquired the absorbance at 450 nm to calculate the results.

### Intracellular ROS measurement

H9c2 cells (5 × 10^7^/L, in logarithmic growth phase) were divided into five groups: a control group, NP group, TP group, NP + quercetin group (pretreatment with quercetin, 10 μM; NP added 24 h later), TP + quercetin group (pretreatment with quercetin, 10 μM; TP added 24 h later) and TP + vehicle group (pretreatment with DMSO, the same volume; TP added 24 h later). Then, all of the cells were incubated for 3 h. The culture fluids were subsequently removed, and intracellular ROS generation was measured as previously described^35^. The intracellular ROS accumulation of H9c2 cells was measured by 2′,7′-dichlorofluorescin diacetate (DCFH-DA)^36^ using an intracellular ROS assay kit. Fluorescence intensity was measured by a laser scanning confocal microscope using an excitation of 488 nm and an emission of 525 nm (at a detection spectrum of 488 nm).

### Measurement of intracellular calcium concentration ([Ca^2+^]_i_)

H9c2 cells were washed three times and were incubated for 1 h with Ca^2+^ solution. Fluo-4 acetoxymethyl ester (Fluo-4/AM, 2 μl)^37^ was added to the cells 30 min before the Ca^2+^ solution was removed. The cells were then cultivated in a Ca^2+^ solution containing BSA for 30 min. Thereafter, quercetin (10 μM) was added to the NP + quercetin group and TP + quercetin group, DMSO was added to the TP + vehicle group, and PBS was added to the NP group and TP group at the same volume. All cells were cultured for 30 min at 37 °C in a 5% CO_2_ atmosphere in the dark. Fluorescence intensity (images analysed with Image-Pro Plus 6.0 software, recorded as F_0_) was measured by laser scanning confocal microscopy using an excitation of 488 nm and an emission of 525 nm. Two minutes later, NP and TP (50% V/V) were added respectively and the fluorescence intensity (recorded as F) was measured. The final fluorescence intensity was recorded as F/F_0_.

### Statistical analysis

Data are presented as means ± standard deviations (means ± SDs). Statistical comparisons were performed using one-way analysis of variance (ANOVA, GraphPad Software, USA). Probabilities of 0.05 or less were considered to be statistically significant.

## Additional Information

**How to cite this article**: Jing, Z. *et al*. Protective Effect of Quercetin on Posttraumatic Cardiac Injury. *Sci. Rep.* **6**, 30812; doi: 10.1038/srep30812 (2016).
